# Neighborhood Environments and Healthy Life Expectancy in Older Adults: A 6-year Longitudinal Cohort Study Based on Data from the Japan Gerontological Evaluation Study

**DOI:** 10.31662/jmaj.2023-0154

**Published:** 2024-06-24

**Authors:** Rikuya Hosokawa, Toshiyuki Ojima, Tomoya Myojin, Jun Aida, Katsunori Kondo, Naoki Kondo

**Affiliations:** 1 Department of Human Health Sciences, Graduate School of Medicine, Kyoto University, Kyoto, Japan; 2 Department of Community Health and Preventive Medicine, Hamamatsu University School of Medicine, Shizuoka, Japan; 3 Department of Public Health, Health Management and Policy, Nara Medical University, Nara, Japan; 4 Department of Oral Health Promotion, Graduate School of Medical and Dental Sciences, Tokyo Medical and Dental University, Tokyo, Japan; 5 Center for Preventive Medical Sciences, Chiba University, Chiba, Japan; 6 Center for Well-being and Society, Nihon Fukushi University, Aichi, Japan; 7 Center for Gerontology and Social Science, National Center for Geriatrics and Gerontology, Aichi, Japan; 8 School of Public Health and Graduate School of Medicine, Kyoto University, Kyoto, Japan

**Keywords:** Neighborhood environment, Healthy life expectancy, Older adults, Physical activity, Walking

## Abstract

**Introduction:**

A well-established association exists between health and neighborhood land use patterns, including parks, roads, and other physical environments, also called the built environment. Previous studies have demonstrated that the built environment influences health, particularly among older populations, because the scope of activities in such populations is limited. Herein, we investigated the association between specific neighborhood environments and the healthy life expectancy of older individuals.

**Methods:**

Data at two time points (2013 and 2019) from the Japan Gerontological Evaluation Study were used in this study. The study comprised a sample of 8,956 residents aged ≥65 years who were not certified for long-term care. Information on the presence or absence of eight types of neighborhood environments was collected using a questionnaire. A multistate life table analysis was conducted to determine the association between perceived neighborhood environments and healthy life expectancy.

**Results:**

Significant differences were observed in the “parks and sidewalks suitable for exercise and walking” category. The group that perceived “parks and sidewalks suitable for exercise and walking” had an approximately 1.2-year longer healthy life expectancy than the group that did not perceive such parks and sidewalks. In addition, individuals who lived within walking distance of a park were more physically active than those who did not.

**Conclusions:**

Safe, walkable neighborhoods with excellent parks may encourage physical activity among older adults and extend their healthy lifespan. Future research is warranted to identify the underlying mechanisms.

## Introduction

Neighborhood environments affect health, and a well-established association exists between health and neighborhood land use practices, including those involving parks, streets, and other built environments. A previous study on the impact of neighborhood environment on health reported an association between built environments and physical activity ^(1)^. Regular physical activity provides numerous health benefits, including prevention, improvement, and reduction in the mortality risk of chronic diseases, such as heart diseases, diabetes, cancer, hypertension, dyslipidemia, obesity, and depression ^(2), (3), (4), (5), (6), (7)^. Thus, physical activity is associated with healthy life expectancy and not merely increased life expectancy. However, majority of the Japanese people do not engage in regular physical activity ^(8)^. Therefore, public health research should explore factors associated with increased levels of physical activity.

Japanese people have a long life expectancy; however, they do not always have a good quality of life or a healthy life. A longer life expectancy is associated with a higher risk of disease and disability before death ^(9), (10)^. Furthermore, aging places growing demands on the economy and social institutions. Hence, effective strategies to promote longer and healthier lives are imperative. Japan’s national health promotion movement, “Healthy Japan 21 (Phase 2),” initiated in 2013, has two main goals: to extend healthy life expectancy and to reduce health disparities ^(11)^. Healthy life expectancy refers to the duration of life spent without any health-related restriction on daily activities.

Neighborhood environments may also be associated with healthy life expectancy. Most previous studies on neighborhood-built environments, physical activity, and health were cross-sectional; moreover, a reverse causality exists that healthy older adults who prefer physical activity choose to live in environments that promote physical activity ^(12)^. Although the specific neighborhood environment associated with healthy life expectancy remains unclear, housing density, road connectivity, and land use mix seem to be important factors that are associated with healthy life expectancy ^(13), (14)^.

Thus, this study determined whether healthy life expectancy in older adults was associated with specific neighborhood environments in Japan. We utilized data from a 6-year follow-up period (2013 to 2019 waves) to identify specific neighborhood environments associated with healthy life expectancy.

## Materials and Methods

### Participants

In this study, we utilized national longitudinal cohort data collected by the Japan Gerontological Evaluation Study (JAGES) in 2013 and 2019 ^(15), (16)^. The study included individuals aged ≥65 years who did not require long-term care and residing in 19 municipalities in 9 of the 47 prefectures in Japan. Self-administered questionnaires were used for the survey. The major survey items included questions regarding the neighborhood environment. Among the 13,662 residents who provided valid responses to the items related to limited activities of daily living in the 2013 survey, 4,846 provided valid responses in the 2019 survey. In addition, we incorporated data from 2,343 participants who were certified as requiring long-term care in the survey conducted in 2019 and 1,767 participants who died between 2013 and 2019. Finally, 8,956 residents were included in the study.

### Exposure: neighborhood environment

The participants were asked about their perceptions toward the neighborhood environment around their homes. The question “How many of the following places are within walking distance (roughly 1 km) of your house?” included five negative aspects, such as “places with graffiti and trash,” “roads and intersections with traffic accident hazards,” “dangerous places to walk alone at night,” and “places where it is difficult to walk (hills and steps),” and four positive aspects, such as “parks and sidewalks,” “attractive views and buildings,” “fresh food stores,” and “houses and facilities for casual drop-ins.” A 5-point scale was used for each aspect with the following responses: “a lot,” “some,” “a little,” “none,” and “do not know.” These five responses were dichotomized into yes (a lot, some) and no (a little, none, do not know). The eight types of perceived neighborhood environments were used as independent variables ^(17)^.

### Outcome: healthy and unhealthy life expectancy

Healthy life expectancy was computed employing the multistate life table method using the SAS Stochastic Population Analysis for Complex Events (SPACE) (SAS Institute Inc., NC, USA) software ^(18)^. The SPACE program comprises SAS, SAS/IML, and SAS/Connect, and its technical details have been provided elsewhere ^(18)^. In our analysis, the Markov transition model of disability and mortality encompassed three states: two nonabsorbed states (nondisability and disability capacity) and one absorbed state (death). Four health status transitions were considered, namely, nondisabled to disabled (disability), disabled to nondisabled (recovery from disability), nondisabled to dead, and disabled to dead. These transitions were based on changes from 2013 (baseline) to 2019 (follow-up), where individuals with no disability were classified as healthy and those with disability as unhealthy. On the basis of this estimated transition rate, the healthy life expectancy at 65 years was calculated for men and women. Considering the aging population, a long-term care insurance (LTCI) system was introduced in Japan ^(19), (20)^. In this study, disability was determined based on the responses to questions regarding disability in daily living at baseline and follow-up, whereas data on the need for LTCI care were collected from municipalities. Death was identified using data from municipalities.

### Ethical statement

This study was approved by the ethics committee of the Nihon Fukushi University (approval number: 13-14), Chiba University (approval number: 2493), and the National Institute for Longevity Sciences (approval number: 992-3). The study protocol adhered to the principles outlined in the Declaration of Helsinki. The participants were informed that their participation was voluntary and that completing the questionnaire, selecting the consent checkbox, and returning the questionnaire via mail implied consent to participate. We considered the return of a completed questionnaire as consent for participation in both the baseline and follow-up surveys. Information on the LTCI certification status and death was also confirmed after obtaining the written consent.

### Statistical analyses

The changes in healthy life expectancy based on the presence or absence of eight types of neighborhood environments were explored using a multistate life table. Furthermore, the association between the neighborhood environment and physical activity was examined using the χ^2^ test, where p <.05 was considered to indicate statistical significance.

## Results

At baseline, the data of 8,956 participants (4,348 men and 4,608 women) were included in the analysis. The perceptions of neighborhood environments across sexes are presented in Table 1. The healthy life expectancies at 65 years in men and women were 16.18 (standard deviation [SD] 0.20) and 16.97 (SD 0.22) years, respectively.

**Table 1.** Perceptions of Neighborhood Environment Types by Sex.

	Men (n = 4348)		Women (n = 4608)		
	n	%	n	%	p
Locations with graffiti or garbage				
Present	1100	25.3	890	19.3	<.001
Absent	3248	74.7	3718	80.7
Roads/crossroads with risk of traffic accidents				
Present	2782	64.0	2721	59.0	<.001
Absent	1566	36.0	1887	41.0
Dangerous places for walking alone at night				
Present	2313	53.2	2611	56.7	<.001
Absent	2035	46.8	1997	43.3
Locations unamenable to walking (hills or steps)				
Present	1479	34.0	1617	35.1	.285
Absent	2869	66.0	2991	64.9
Access to parks and sidewalks				
Present	3245	74.6	3240	70.3	<.001
Absent	1103	25.4	1368	29.7
Fascinating views or buildings				
Present	1563	35.9	1822	39.5	<.001
Absent	2785	64.1	2786	60.5
Access to fresh food stores				
Present	3313	76.2	3310	71.8	<.001
Absent	1035	23.8	1298	28.2
Houses or facilities where you feel free to drop in				
Present	1531	35.2	1692	36.7	.137
Absent	2817	64.8	2916	63.3

Table 2 and 3 present the association between the neighborhood environment within walking distance (generally within 1 km) of men’s and women’s homes and healthy life expectancies, respectively. Significant differences were observed in the item “parks and sidewalks suitable for exercise and walking.” Participants who perceived the existence of “parks and sidewalks suitable for exercise and walking” had longer healthy life expectancies―longer by 1.31 and 1.32 years in men and women, respectively―than those who did not.

**Table 2.** Association between a Neighborhood Environment within Walking Distance (Generally within 1 km) of Men’s Homes and Healthy Life Expectancy.

	Years	95% CI	
Locations with graffiti or garbage		
Present	16.38	15.56	17.20
Absent	16.06	15.59	16.53
Roads/crossroads with risk of traffic accidents		
Present	16.25	15.75	16.74
Absent	16.17	15.50	16.84
Dangerous places for walking alone at night		
Present	16.37	15.83	16.90
Absent	15.89	15.33	16.46
Locations unamenable to walking (hills or steps)		
Present	15.90	15.25	16.56
Absent	16.26	15.78	16.74
Access to parks and sidewalks		
Present	**16.50**	**16.05**	**16.96**
Absent	**15.19**	**14.43**	**15.95**
Fascinating views or buildings		
Present	16.51	15.85	17.17
Absent	15.89	15.41	16.37
Access to fresh food stores		
Present	16.47	16.06	16.89
Absent	15.38	14.52	16.24
Houses or facilities where you feel free to drop in		
Present	16.16	15.49	16.83
Absent	16.26	15.77	16.75

Bold numbering indicates a significant difference, with men who had access to parks having significantly longer life expectancy than men who did not have access to parks. CI, confidence interval

**Table 3.** Association between a Neighborhood Environment within Walking Distance (Generally within 1 km) of Women’s Homes and Healthy Life Expectancy.

	Years	95% CI	
Locations with graffiti or garbage		
Present	17.05	16.14	17.96
Absent	16.99	16.51	17.48
Roads/crossroads with risk of traffic accidents		
Present	17.12	16.56	17.69
Absent	16.80	16.13	17.47
Dangerous places for walking alone at night		
Present	17.25	16.71	17.78
Absent	16.61	15.93	17.29
Locations unamenable to walking (hills or steps)		
Present	16.89	16.22	17.56
Absent	17.12	16.58	17.66
Access to parks and sidewalks		
Present	**17.39**	**16.88**	**17.89**
Absent	**16.07**	**15.27**	**16.86**
Fascinating views or buildings		
Present	17.42	16.68	18.15
Absent	16.74	16.16	17.31
Access to fresh food stores		
Present	17.00	16.56	17.45
Absent	16.46	15.62	17.29
Houses or facilities where you feel free to drop in		
Present	17.67	17.01	18.33
Absent	16.67	16.10	17.23

Bold numbering indicates a significant difference, with women who had access to parks having significantly longer life expectancy than women who did not have access to parks. CI, confidence interval

Furthermore, when the association between “parks and sidewalks suitable for exercise and walking” and physical activity was examined (Supplementary Tables 1 and 2), participants who perceived the existence of “parks and sidewalks suitable for exercise and walking” were more likely to be physically active.

## Discussion

This study investigated the association between perceived neighborhood environments and healthy life expectancy using data from the JAGES 2013 to 2019 wave. It focused on the Japanese population aged 65 years and above, aiming to contribute to the development of a healthy and long-lived society. When examining the association between the neighborhood environment and healthy life expectancy, significant differences were observed in the variable “parks and sidewalks suitable for exercise and walking.” Participants who perceived “parks and sidewalks suitable for exercise and walking” had a longer healthy life expectancy than those who did not.

Significant sex differences were observed in the perception of buildings in the neighborhood. This could be attributed to the underlying differences in each sex’s interest in and use of the building. Variables associated with a negative neighborhood environment, including areas with graffiti and rubbish, streets and intersections posing traffic accident risks, places considered dangerous to walk alone at night, and places considered difficult to walk in (slopes and steps), were included as options but were not associated with healthy life expectancy. Contrarily, the neighborhood environment variable that improved healthy life expectancy in this study was access to parks and sidewalks. This result may be overinterpreted because active individuals are likely to perceive their environment differently than inactive individuals. Previous studies have demonstrated that older adults living near parks exercise more frequently ^(21), (22)^. In the present study, perceived proximity to parks was also positively associated with physical activity. A decline in physical activity among older adults is a risk factor for long-term care need and is an important public health concern. Furthermore, lack of physical activity and exercise contributes to declining physical health.

This study has the following strengths: First, it is a large survey study encompassing 9 prefectures and 22 municipalities in Japan. Second, data were collected from two time points, accounting for time factors. However, this study also has some limitations that need to be acknowledged. First, this study was based on a subjective assessment of the characteristics of the neighborhood environment. However, incorporating objective indicators complementing a subjective evaluation may enable a more detailed assessment of the neighborhood environment. Nevertheless, subjective assessments tend to more closely reflect the extent to which people perceive their environment and the reality in which they navigate that environment.

This study included individuals aged ≥65 years who did not require long-term care and residing in 19 municipalities in 9 of the 47 prefectures in Japan. Healthy life expectancy was not found to be associated with the negative aspects of the neighborhood environment, but longer healthy life expectancy was associated with the positive aspect of “access to parks and sidewalks.” Our findings confirm that older people who live closer to parks are also more likely to be physically active. Safe, walkable neighborhoods with excellent parks may promote physical activity among older adults and extend their healthy lifespan.

## Article Information

### Conflicts of Interest

None

### Sources of Funding

This work was supported by Health Labor Sciences Research Grants grant number 22FA1010.

### Acknowledgement

We are grateful to the Health Labor Sciences Research Council for funding this study.

### Author Contributions

NK was responsible for acquiring funds. RH and TO were responsible for the research design and investigation, methodology, resources, software used, validation and visualization of data, and writing the original draft of the manuscript. TO was responsible for supervising the study. NK was responsible for the project administration. TM, JA, KK, and NK were responsible for reviewing and editing the manuscript. All authors have approved the final version of the manuscript.

### ORCID iD

Rikuya Hosokawa: https://orcid.org/0000-0003-4239-8494

### Approval by Institutional Review Board (IRB)

Nihon Fukushi University (approval number: 13-14), Chiba University (approval number: 2493), and National Institute for Longevity Sciences (approval number: 992-3).

## Supplement

Supplementary Materials
