# Supplementary material for: Neighborhood Environments and Healthy Life Expectancy in Older Adults: A 6-year Longitudinal Cohort Study Based on Data from the Japan Gerontological Evaluation Study
Source: JMA J. 2024 Jun 24;7(3):328–33. doi: 10.31662/jmaj.2023-0154 (PMC11301004; doi:10.31662/jmaj.2023-0154)
Supplement: Supplementary Materials [file 2433-3298-7-3-0328-s001.pdf]

Neighborhood environments and healthy life expectancy in older adults: a six-year longitudinal cohort study based on data from the Japan Gerontological Evaluation Study

Supplementary Materials

Supplementary Table 1. Association between neighborhood park availability and physical activity in men

	Physically active		Physically inactive		
	n	%	n	%	p
Strenuous exercise					
Park in the neighborhood	613	20.49	2378	79.51	<.0001
No park in the neighborhood	149	15.62	805	84.38	
Moderate activity					
Park in the neighborhood	1949	62.57	1166	37.43	<.0001
No park in the neighborhood	525	53.19	462	46.81	
Light activity					
Park in the neighborhood	2041	67.36	989	32.64	<.0001
No park in the neighborhood	525	55.03	429	44.50	

Note: Excluding no answers

Supplementary Table 2. Association between neighborhood park availability and physical activity in women

	Physically active		Physically inactive		
	n	%	n	%	p
Strenuous exercise					
Park in the neighborhood	560	19.89	2255	80.11	<.0001
No park in the neighborhood	155	14.21	936	85.79	
Moderate activity					
Park in the neighborhood	1861	62.49	1117	37.51	<.0001
No park in the neighborhood	588	51.76	548	48.24	
Light activity					
Park in the neighborhood	2670	87.51	381	12.49	<.0001
No park in the neighborhood	886	75.86	282	24.14	

Note: Excluding no answers
